# Associations of diet composition and quality with continuous glucose monitor-derived glycemic metrics in a community-based cohort

**DOI:** 10.1016/j.ajcnut.2025.07.026

**Published:** 2025-07-31

**Authors:** Bahar Bakhshi, Naznin Sultana, Honghuang Lin, David Fei, Valeria Vallejo, Abigail Gatanti, Devin W Steenkamp, Joanne M Murabito, Nicola M McKeown, Maura E Walker, Nicole L Spartano

**Affiliations:** 1 Section of Endocrinology, Diabetes, Nutrition, and Weight Management, Boston University Chobanian and Avedisian School of Medicine (BUCASM) and Boston Medical Center, Boston, MA, United States; 2 Department of Biostatistics, Boston University School of Public Health (BUSPH), Boston, MA, United States; 3 Department of Medicine, University of Massachusetts Chan Medical School, Worcester, MA, United States; 4 Department of Medicine, Section of General Internal Medicine, BUCASM and Boston Medical Center, Boston, MA, United States; 5 Boston University’s and National Heart, Lung, and Blood Institute’s Framingham Heart Study, Framingham, MA United States; 6 Department of Health Sciences, Sargent College of Health and Rehabilitation Sciences, Boston University, Boston, MA, United States

**Keywords:** Continuous glucose monitoring, glycemic variability, carbohydrate quality, diet quality, type 2 diabetes

## Abstract

**Background:**

Continuous glucose monitors (CGMs) offer real-time assessment of glucose concentrations, providing an opportunity to understand the determinants of glycemic variability.

**Objectives:**

We aimed to cross-sectionally evaluate the associations between carbohydrate substitution, indices of diet, and carbohydrate quality with CGM-derived measures in individuals without diabetes.

**Methods:**

Participants from the Framingham Heart Study, with ≥3 days of CGM data and ≥2 days of dietary records, were included in this analysis. CGM-derived glycemic traits were calculated using the “cgmanalysis” R package. Multivariable linear regression models were used to derive beta coeffcients and standard erros (SE) from the relationships of carbohydrate substitution, carbohydrate quality (fiber intake and carb-to-fiber ratio), and indices of diet quality (healthy eating index, alternate healthy eating index, dietary approaches to stop hypertension and alternate Mediterranean diet score) with CGM-derived measures among all participants and stratified by glycemic status. Least-squares means were estimated to visualize adjusted group differences of percent time spent above 140 mg/dL across quartiles of diet and carbohydrate quality metrics.

**Results:**

We included 677 individuals in this analysis (56.9% with normoglycemia, 59.4% female, mean age 60.1 y, mean glucose 117.7 mg/dL). Overall, higher diet and carbohydrate quality were associated with favorable CGM-derived measures. Replacing 5% energy intake from protein with the equivalent from carbohydrate was associated with 0.97 mg/dL higher CGM mean glucose (SE = 0.47; *P* = 0.04). The associations of diet quality with glycemic variability were typically more pronounced among those with normoglycemia, but for those with prediabetes, consuming a diet with >1 g fiber for every ∼9 g carbohydrates was associated with 7%–10% lower time spent >140 mg/dL compared with higher carb-to-fiber ratios (*P*-trend < 0.001).

**Conclusions:**

Our findings demonstrate that carbohydrate intake and quality, in addition to overall diet quality, are associated with dynamic fluctuations in glucose concentrations. Future prospective analysis should examine whether glycemic variability mediates the association between diet and incident type 2 diabetes.

## Introduction

Type 2 diabetes (T2D) is a phenotypically heterogeneous disease [1], yet the risk of T2D is largely attributed to modifiable lifestyle factors, including poor diet quality, physical inactivity/sedentary behavior, tobacco smoking, alcohol consumption, and inadequate sleep, which are all associated with excess body weight [[2], [3], [4], [5]]. The role of poor diet quality in relation to insulin resistance and T2D incidence has been extensively studied [6,7]. In observational studies and randomized controlled trials, a diet with low glycemic index [8,9], as well as adherence to dietary patterns such as the Mediterranean-style diet [10] and the dietary approaches to stop hypertension (DASH) [11,12] has been associated with better glycemic metrics among individuals with or without T2D. However, these studies have relied on “snapshot” clinical markers of blood glucose, including fasting plasma glucose, hemoglobin A1c (HbA1c), and 2-h postprandial glucose, which overlook the dynamic fluctuations in glucose concentrations that may be indicative of the true glycemic burden [13,14].

With the growing availability of accurate continuous glucose monitors (CGMs), it is now possible to assess the magnitude, duration, and frequency of variations in interstitial glucose, and its association with diet composition and quality [15]. To date, however, few observational studies have related diet to CGM data. A recent cross-sectional investigation, involving >7000 adults without diabetes, reported a significant correlation between median percent macronutrient intake and various CGM-derived glycemic measures [16]. In another small cross-sectional study of 94 individuals, percent carbohydrate, protein, and energy intake (EI) were correlated with glycemic variability only among those with impaired glucose tolerance but not normoglycemic individuals [17]. However, it remains unclear whether different aspects of diet, other than macronutrient quantity, are associated with CGM-derived glycemic traits, after considering demographic and lifestyle factors.

In this analysis, we aimed to cross-sectionally assess the relationship between carbohydrate quality, diet quality metrics, in addition to carbohydrate substitution with key CGM-derived glycemic traits within the Framingham Heart Study (FHS). In a secondary analysis, we examined whether the association between dietary factors and CGM-derived glycemic traits differs according to normoglycemia or prediabetes status. Results from this study will contribute to understanding the determinants of glycemic variability and facilitate establishing dietary recommendations for glycemic control and the prevention of T2D.

## Methods

### Study sample

The details of FHS are described elsewhere [18]. For the current analysis, we included data from the FHS third generation (*n* = 1156), new offspring (*n* = 22), and omni 2 (*n* = 113) participants, who attened the first year of their fourth examination cycle (September 2022 to September 2023). During their examination at the FHS center, all participants were offered to wear a Dexcom G6 pro CGM for 10 days on their arm or abdomen. Although while wearing the device, participants did not have access to their glucose data (blinded setting); however, they received a report with this information afterward. Additionally, participants were asked to complete dietary records for 3 days and wear a physical activity tracker for 7 days during the time they were wearing the CGM. Details of participant enrollment are included in Supplemental Figure 1. All FHS study protocols and procedures were conducted according to the guidelines of the Declaration of Helsinki and approved by the institutional review board for human research at Boston University. All participants provided written consent prior to participation in the study.

We excluded individuals who declined to wear a CGM device (*n* = 108), did not return the CGM device to the FHS center (*n* = 76), had CGM devices with no transmitted data (*n* = 29), had CGM devices with no data on complete days (*n* = 20), and had CGM devices with only 1 or 2 d of transmitted data (*n* = 27). Further, we excluded participants who did not complete any dietary records (*n* = 186), had only 1 day of dietary record (*n* = 60), had implausible EI (females; <600, >4400 kcal/d, males; <650, >5700 kcal/d), reported extreme portions of a food consumed at 1 eating occasion (*n* = 1) [19]. We further excluded participants who had diabetes defined through the following criteria: HbA1c ≥ 6.5%, fasting glucose ≥126mg/dL, self-reported or prior history of diabetes, or taking glucose-lowering medications (*n* = 81). Lastly, we excluded those who had missing information on important covariates (*n* = 24). The final eligible sample size was 677, including 385 individuals with normoglycemia (HbA1c < 5.7% and fasting glucose <100 mg/dL) and 292 individuals with prediabetes (HbA1c 5.7–6.4% or fasting glucose 100–125 mg/dL) (Supplemental Figure 1). We have included participant characteristics of those who did not complete any dietary records according to their glycemic status in Supplemental Table 1.

### Dietary assessment

Dietary data were collected through the automated self-administered 24-h dietary assessment (ASA24), a web-based tool developed by the National Cancer Institute [20]. Participants were prompted to complete 3 days of dietary records, including 2 weekdays and 1 weekend. The average intake of foods and nutrients was estimated from ≥2 days of dietary records. Macronutrient intake was defined as a percent of total EI. Fiber intake, defined as total grams of fiber per 1000 kcal, and carb-to-fiber ratio, defined as the ratio of total grams of carbohydrates to total grams of fiber, were used as a measure of carbohydrate quality [21]. To assess diet quality, the healthy eating index 2020 (HEI-2020), alternate HEI 2010 (AHEI-2010), DASH, and alternate Mediterranean diet score (aMED) were included in the analysis. Details on calculations, in addition to a description of the scores and their components, are included in Supplemental Tables 2–4. The HEI-2020 assesses adherence to 13 components corresponding to the 2020–2025 dietary guidelines for Americans, scores ranging from 0 to 100, with 100 indicating complete alignment with the dietary guidelines for Americans [22]. The AHEI consists of foods and nutrients predictive of lower chronic disease risk [23], is based on 11 components [24], and the revised AHEI-2010 scores range from 0 to 100 (with the *trans* fat category removed from the scoring). The DASH index is composed of 8 components and utilizes a sex-specific scoring system to rank individuals according to the cohort-specific quintiles, with scores ranging from 8 to 40 [25]. The aMED evaluates adherence to the Mediterranean diet among the United States population [26]. The aMED includes 9 components and categorizes individuals based on cohort- and sex-specific median, and scores range from 1 to 9 [26].

### Outcome definition

The Dexcom pro CGM sensor records interstitial glucose value every 5 min for the duration the sensor is worn (average wear time in hours was 200). CGM-derived measures of glycemic variability were calculated with the *cgmanalysis* R package, which is a standardized, free, and open-source package for generating CGM summary measures [27]. A brief description of CGM-derived glycemic metrics used in this study is provided in Table 1. To further elucidate the relationship between CGM-derived glycemic traits and diet, we chose 6 CGM summary measures representative of mean glucose, high glucose concentrations, and glucose variability based on previous literature [16]. We intended to include both commonly used metrics and less studies ones, including mean glucose, coefficient of variation (CV), J-index, mean amplitude of glycemic excursions (MAGE), continuous overall net glycemic action (over 1 hour) (CONGA-1), mean of daily differences (MODD), and percent of time spent above 140 mg/dL. Each summary measure offers valuable information on different aspects of glycemic variability. Mean glucose reflects average glucose concentration during the entire device wear period, CV is a standardized measure of variability, providing a normalized measure of dispersion from the mean, and J-index integrates mean glucose and SD to provide a measure of glycemic control [28,29]. MAGE and CONGA-1 are both measures of within-day variability, whereas MODD is a measure of between-day glycemic variability [30,31]. The percent of time spent above 140 mg/dL is calculated by summing the total monitored time during which an individual’s interstitial glucose concentrations exceed 140 mg/dL [32].

**TABLE 1:** Distribution and definition of key continuous glucose monitor-derived measures of glycemic variability among all participants and according to glycemic status.

CGM metrics	Total population	Individuals with normoglycemia	Individuals with prediabetes	Definition
*n* Participants	677	385	292	N/A
Average CGM wear (*n* days)^1^	8.3	8.3	8.3	N/A
Mean glucose (mg/dL)				The average glucose concentration during the entire device wear period.
Mean (SD)	117.7 (13.1)	113.7 (11.2)	122.9 (13.7)	
Median [IQR]	116.83 [16.0]	112.9 [15.6]	121.4 [17.1]	
CV (%)				Measure of glycemic variability relative to the mean. CV (%) = (mean/SD) ×100
Mean (SD)	15.2 (2.9)	15 (2.9)	15.5 (2.9)	
Median [IQR]	15.08 [3.8]	14.1 [3.7]	15.4 [3.8]	
J-index				Measures quality of glycemic control by integrating mean and SD [35]. J-index = 0.001 × (mean + SD)^2^
Mean (SD)	19.1 (4.5)	17.7 (3.5)	21 (5.0)	
Median [IQR]	18.5 [5.4]	17.7 [3.5]	20.3 [6.1]	
MAGE (mg/dL)				Measure of within-day or intraday glycemic variability. Represents the average magnitude of glycemic excursions between consecutive peaks (highest points) and nadirs (lowest points) during the specified wear period, only taking into account the changes in blood glucose that are >1 SD of the blood glucose for a 24-h period [30].
Mean (SD)	40.4 (10.5)	38.4 (9.6)	43.2 (11.0)	
Median [IQR]	38.0 [12.5]	36.6 [11.5]	42.1 [12.2]	
CONGA-1 (mg/dL)				Measure of within-day or intraday glycemic variability. Represents the differences between consecutive blood glucose readings taken at 1-h intervals across all days [36].
Mean (SD)	21.3 (4.7)	22.4 (4.7)	22.4 (4.7)	
Median [IQR]	20.8 [6.4]	20.5 [5.7]	22.4 [6.2]	
MODD (mg/dL)				Measure of between-day or day-to-day glycemic variability. Represents the mean of absolute differences in glucose concentrations at the same time point on consecutive days [37].
Mean (SD)	17.6 (3.9)	16.6 (3.3)	18.9 (4.3)	
Median [IQR]	16.9 [4.6]	16. 3 [4.1]	18.2 [5.5]	
Time >140 mg/dL (%)				Percentage of total monitored time during which an individual’s interstitial glucose concentrations exceed 140 mg/dL [32].
Mean (SD)	16.1 (14.9)	11.6 (9.8)	22.1 (18.2)	
Median [IQR]	11.6 [15.2]	9.2 [11.2]	16.8 [20.6]	

Abbreviations: CGM, continuous glucose monitor; CONGA-1, continuous overall net glycemic action (measured over 1 hour); CV, coefficient of variation; IQR, interquartile range; MAGE, mean amplitude of glycemic excursion; MODD, mean of daily difference; SD, standard deviation; N/A, Not applicable.

1 After removing the first and partial last day.

### Covariates definition

Potential confounders of the relationship between carbohydrate substitution, metrics of carbohydrate and diet quality, and CGM-derived measures of glycemic variability were included as covariates in our analysis based on the prior literature: age (years), sex (male/female); EI (kcal/day); current smoker (reported smoking regularly in the last year); education level (no high school degree, high school degree, some college education, college graduate); alcohol consumption (grams/day); pharmacologic treatment of hypertension (yes/no); pharmacologic treatment of dyslipidemia (yes/no); average number of steps per day (obtained from Fitbit); BMI [weight (kg) divided by height (m) squared (kg/m^2^)].

### Statistical analysis

The characteristics of participants according to glycemic status were reported as mean (SD) for continuous variables and *n* (%) for categorical variables. Pairwise correlation coefficients were used to evaluate the correlations between key CGM-derived measures of glycemic variability. Macronutrient intakes were calculated using the nutrient density approach [33] and were expressed as 5% change from EI. Carbohydrate quality and diet quality measures were standardized to a mean of 0 and an SD of 1. Independent associations were evaluated using multivariable linear regression. In all analysis, model 1 was adjusted for age, sex, and EI. Model 2 was age, sex, EI, education, smoking status, alcohol consumption, hypertension medication, cholesterol-lowering medication, and average steps per day. Model 3 was additionally adjusted for BMI. We evaluated how 5% increase in energy from carbohydrates at the expense of protein or fat is associated with CGM-derived measures, through substitution analysis with mutually adjusting for protein or fat. Nutrient substitution models are used to evaluate the potential health implications of replacing one macronutrient with another, while holding total EI constant [34]. Effect modification of glycemic status (normoglycemia or prediabetes) was tested by including the cross-product term with each carbohydrate quality and diet quality variable in the corresponding regression models. We tested for a linear trend of the percent of time spent >140 mg/dL across quartile categories of each respective dietary exposure. For each dietary exposure, the median value across quartile categories was modeled as a continuous variable in linear regression models with log-transformed percent of time spent >140 mg/dL as the outcome. *P* values from the test for linear trend are depicted with least-squares means of time spent >140 mg/dL across quartile categories. All statistical tests were considered 2-sided. To address multiple hypotheses, we used the Benjamini-Hochberg procedure, and statistical significance was considered as a false discovery rate corrected *P* value of <0.05. Similarly, *P* values for interactions were set to a significance level of false discovery rate corrected <0.2. All analyses were performed in R version 4.3.1.

## Results

We included 677 individuals in this analysis, of whom 56.9% had normoglycemia and 59.4% were female. The mean age was 60.1 (SD: 8.4), and the mean BMI was 27.7 (SD: 5.1). Individuals with prediabetes were slightly older, had a lower proportion of females, and were college degree holders. Additionally, a higher proportion of individuals with prediabetes took hypertension and lipid-lowering medication, had a higher average BMI, and took fewer steps per day. In terms of dietary intake, individuals with prediabetes had a slightly higher EI and absolute intakes of all macronutrients (Table 2).

**TABLE 2:** Characteristics of participants according to glycemic status.

	Total population	Individuals with normoglycemia	Individuals with prediabetes
*n* Participants	677	385	292
Characteristics^1^			
Sex, female	402(59.4)	260 (67.5)	142 (48.6)
Age, y	60.1 (8.4)	58.9 (8.7)	61.6 (7.7)
Race, White, or Caucasian	618 (91.3)	355 (92.2)	263 (90.0)
Education, college degree	482 (71.2)	291 (75.6)	191 (56.4)
Current smoker	21 (3.1)	11 (3.0)	10 (3.4)
HbA1c	5.3 (0.3)	5.2 (0.3)	5.5 (0.2)
Hypertension medication	160 (23.6)	60 (15.6)	100 (34.2)
Lipid-lowering medication	176 (25.9)	78 (20.3)	98 (33.6)
BMI, kg/m^2^	27.7 (5.1)	26.5 (4.8)	29.2 (5.1)
Steps, average/d	9648 (4043)	9771 (3928)	9488 (4192)
Dietary intake^1^			
Total energy, kcal/d	1931 (652)	1867 (627)	2017 (675)
Carbohydrate, g/d	201.4 (78.8)	195.4 (74.5)	209.2 (83.7)
Protein, g/d	82.1 (30.6)	79.1 (32,4)	86.5 (32.8)
Fat, g/d	82.2 (32.7)	78.9 (30.3)	86.4 (30.5)
Fiber, g/d	18.5 (9.3)	18.4 (9.4)	18.6 (9.3)
Total alcohol, g/d	12.8 (21.2)	12.8 (21.8)	12.8 (20.5)
Carbohydrate, % of EI	41.8 (8.9)	42.1 (8.6)	41.4 (9.3)
Protein, % of EI	17.3 (4.4)	17.2 (4.3)	17.6 (4.5)
Fat, % of EI	38.1 (7.3)	37.9 (7.5)	38.5 (7.1)
Fiber, g/1000 kcal	9.8 (4.2)	10.2 (4.3)	9.4 (3.9)
Carb-to-fiber ratio	12.1 (5.0)	11.9 (5.1)	12.4 (4.8)
HEI-2020, 0–100	61.1 (13.6)	61.7 (13.8)	60.4 (13.3)
AHEI-2010, 0–100	52.7 (13.2)	53.5 (13.1)	51.7 (13.2)
DASH, 8–40	24.4 (5.9)	24.6 (5.9)	24.1 (5.9)
aMED, 0–9	4.05 (1.64)	4.11 (1.6)	4.1 (1.7)

Abbreviations: AHEI-2010, alternate healthy eating index; aMED, alternate Mediterranean diet; BMI, body mass index; DASH, dietary approaches to stop hypertension; EI, energy intake; HbA1c, hemoglobin A1c; HEI-2020, healthy eating index-2020; SD, standard deviation.

1 Data are presented as mean (SD) for continuous variables and *n* (%) for categorical variables.

**FIGURE 1:**
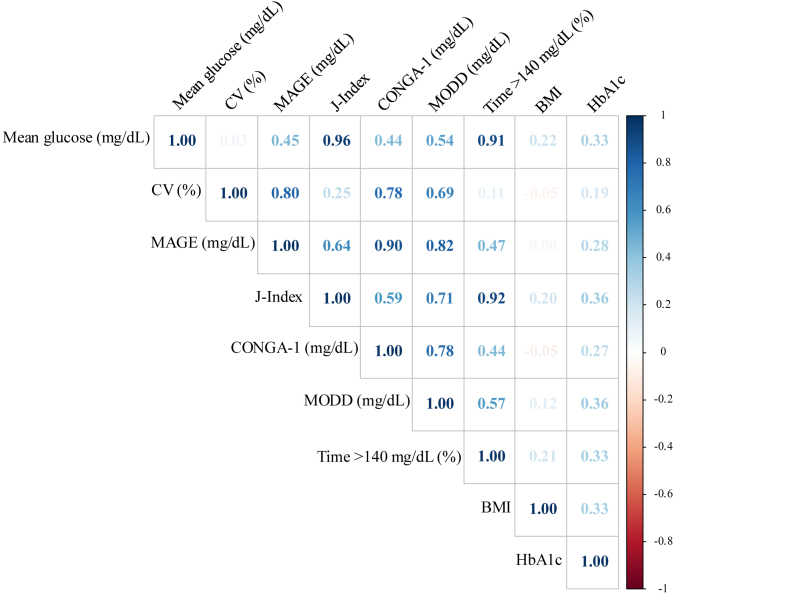
Unadjusted pairwise correlations of the selected CGM-derived measures with each other, in addition to BMI and HbA1c among all participants (*n* = 677). BMI, body mass index; CGM, continuous glucose monitor; CONGA-1, continuous overall net glycemic action (over 1 hour); CV, coefficient of variation; HbA1c, hemoglobin A1c; MAGE, mean amplitude of glycemic excursion; MODD, mean of daily difference.

Figure 1 illustrates unadjusted pairwise correlations of the selected CGM-derived measures of glycemic variability with each other, in addition to BMI and HbA1c. Except for J-index and percent of time spent >140 mg/dL, all other CGM summary measures were weakly or moderately correlated with mean glucose (mg/dL) [CV (%); *r* = 0.03, MAGE; *r* = 0.45; J-index; *r* = 0.96; CONGA-1; *r* = 0.44, MODD; *r* = 0.54, % time spent >140 mg/dL; *r* = 0.91]. All CGM summary measures were weakly correlated with BMI and HbA1c [BMI: mean glucose; *r* = 0.22, CV (%); *r* = –0.05, MAGE; *r* = 0, J-index; *r* = 0.2, CONGA-1; *r* = –0.05, MODD; *r* = 0.12, % time spent >140 mg/dL; *r* = 0.33 and HbA1c: mean glucose; *r* = 0.21, CV (%); *r* = 0.19, MAGE; *r* = 0.28, J-index; *r* = 0.36, CONGA-1; *r* = 0.27, MODD; *r* = 0.36, % time spent >140 mg/dL; *r* = 0.33]. In our population, mean glucose was 117.7 mg/dL (SD: 13.1), and individuals with prediabetes presented higher average values for all 7 CGM-derived summary measures (Table 1 [30,32,35–37]).

**FIGURE 2:**
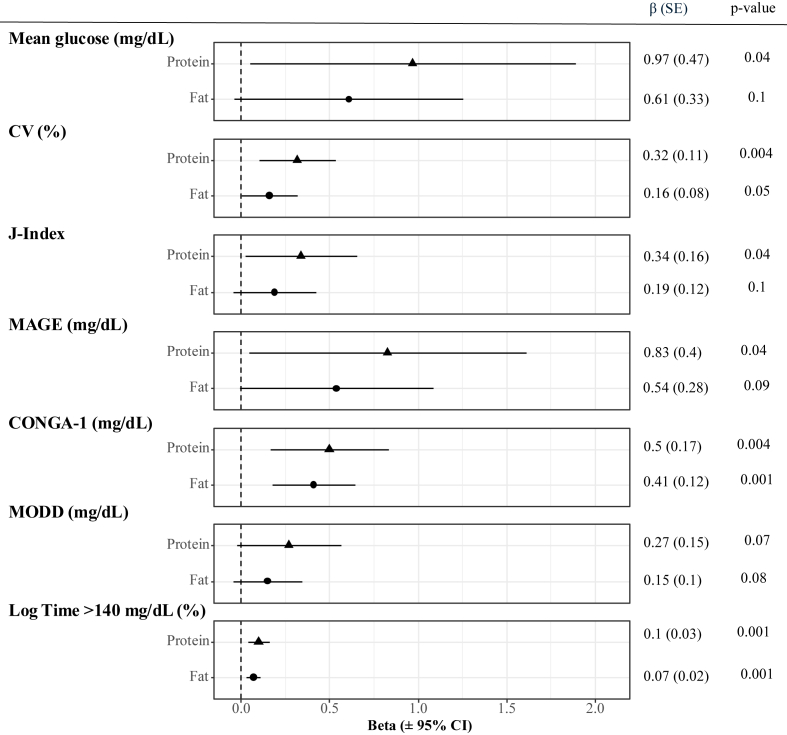
Multivariable linear associations^1^ between isocaloric substitution of carbohydrate (per 5% increment of energy) and CGM-derived measures of glycemic variability among all participants (*n* = 677). BMI, body mass index; CGM, continuous glucose monitor; CI, confidence interval; CONGA-1, continuous overall net glycemic action (over 1 hour); CV, coefficient of variation; MAGE, mean amplitude of glycemic excursion; MODD, mean of daily difference; SE, standard error. ^1^Model was adjusted for age, sex, energy intake, smoking status, education level, alcohol intake, hypertension medication, cholesterol-lowering medication, average steps, BMI, mutual adjustment for protein or fat. *P* values were false discovery rate-adjusted.

Results from the isocaloric substitution models indicated positive associations of substituting carbohydrate (as percentage from EI) with protein and all key CGM-derived measures of glycemic variability, except for MODD (Figure 2). For instance, replacing 5% EI from protein with the equivalent from carbohydrate was associated with 0.97 mg/dL higher mean glucose, after adjusting for potential confounders (SE = 0.47, *P* = 0.04). With regards to carbohydrate substitution for fat, significant associations were only observed for CONGA-1. Replacing 5% EI from fat with the equivalent from carbohydrate was only associated with a 0.41 higher CONGA-1 (SE = 0.12, *P* = 0.001), and a borderline positive significant association was detected for CV [β (SE): 0.16 (0.08), *P* = 0.05].

We found significant associations between metrics of carbohydrate quality and most CGM-derived summary measures (Table 3). After controlling for potential confounders, a 1 SD increase in fiber intake (g/1000 kcal) was associated with 1.76 mg/dL lower mean glucose concentrations (SE: 0.51, *P* = 0.001). The association between fiber intake (g/1000 kcal) with mean glucose persisted after further adjustment for BMI [β (SE): –1.42 (0.53), *P* = 0.01]. In our fully adjusted models, fiber intake (g/1000 kcal) was inversely associated with J-index, CONGA-1, and MODD (*P* < 0.2), whereas no significant associations were observed for CV (%) and MAGE. Additionally, the carb-to-fiber ratio was positively associated with all 7 CGM-derived summary measures. Per 1 SD increase in carb-to-fiber ratio, mean glucose was 1.80 mg/dL (SE = 0.49, *P* = 0.001) higher, which remained significant after additional adjusting for BMI [β (SE): 1.58 (0.49), *P* = 0.004].

**TABLE 3:** Multivariable linear associations of carbohydrate quality metrics (per 1 SD increment) with continuous glucose monitor-derived measures of glycemic variability among all participants (*n* = 677).

Glycemic metrics		Fiber intake (g/1000 kcal)		Carb-to-fiber ratio	
		β (SE)	*P* value^4^	β (SE)	*P* value
Mean glucose (mg/dL)	Model 1^1^	–1.97 (0.49)	<0.001	2.08 (0.49)	<0.001
	Model 2^2^	–1.76 (0.51)	0.002	1.80 (0.49)	0.001
	Model 3^3^	–1.42 (0.53)	0.009	1.58 (0.49)	0.003
CV (%)	Model 1	–0.06 (0.11)	0.61	0.37 (0.11)	0.003
	Model 2	–0.06 (0.12)	0.61	0.34 (0.12)	0.006
	Model 3	–0.10 (0.12)	0.41	0.37 (0.12)	0.004
J-index	Model 1	–0.74 (0.17)	<0.001	0.81 (0.17)	<0.001
	Model 2	–0.64 (0.18)	0.001	0.69 (0.17)	<0.001
	Model 3	–0.53 (0.18)	0.006	0.63 (0.17)	0.001
MAGE (mg/dL)	Model 1	–0.73 (0.41)	0.07	1.39 (0.4)	0.002
	Model 2	–0.63 (0.44)	0.16	1.28 (0.41)	0.004
	Model 3	–0.71 (0.45)	0.12	1.32 (0.42)	0.004
CONGA-1 (mg/dL)	Model 1	–0.35 (0.18)	0.05	0.74 (0.18)	<0.001
	Model 2	–0.41 (0.52)	0.04	0.73 (0.18)	<0.001
	Model 3	–0.48 (0.2)	0.02	0.78 (0.18)	<0.001
MODD (mg/dL)	Model 1	–0.59 (0.15)	<0.001	0.77 (0.15)	<0.001
	Model 2	–0.51 (0.16)	0.004	0.7 (0.15)	<0.001
	Model 3	–0.45 (0.16)	0.009	0.67 (0.15)	<0.001
Log Time >140 mg/dL (%)	Model 1	–0.14 (0.03)	<0.001	0.19 (0.03)	<0.001
	Model 2	–0.13 (0.03)	<0.001	0.17 (0.03)	<0.001
	Model 3	–0.12 (0.04)	<0.001	0.16 (0.03)	<0.001

Abbreviations: CONGA-1, continuous overall net glycemic action (measured over 1 hour); CV, coefficient of variation; MAGE, mean amplitude of glycemic excursion; MODD, mean of daily difference; SD, standard deviation; SE, standard error.

1 Model 1 was adjusted for age, sex, and energy intake.

2 Model 2 was adjusted for model 1 plus smoking status, education level, alcohol intake, hypertension medication, cholesterol-lowering medication, and average steps per day.

3 Model 3 was adjusted for model 2 plus BMI.

4 False discovery rate-adjusted *P* values.

Similarly, metrics of overall diet quality were inversely associated with CGM-derived summary measures (Table 4). After adjusting for lifestyle factors, a 1 SD increase in HEI-2020 was associated with 1.72 mg/dL lower mean glucose (SE = 0.50, *P* < 0.001), which remained significant after further adjustment for BMI [β (SE): –1.37 (0.51), *P* = 0.01]. In terms of magnitude of associations, higher adherence to HEI-2020 and DASH patterns resulted in slightly greater reductions in mean glucose concentrations [DASH; –1.62 (0.51), *P* = 0.002], which remained significant after adjusting for BMI [β (SE): –1.24 (0.52), *P* = 0.02]. In our fully adjusted models, all metrics of diet quality were associated with lower CV (%), J-index, MAGE, CONGA-1, MODD, and log percent of time spent >140 mg/dL.

**TABLE 4:** Multivariable linear associations of diet quality indices (per 1 SD increment) with continuous glucose monitor-derived measures of glycemic variability among all participants (*n* = 677).

Glycemic metrics		HEI-2020		AHEI-2010		DASH		aMED	
		β (SE)	*P* value^4^	β (SE)	*P* value	β (SE)	*P* value	β (SE)	*P* value
Mean glucose (mg/dL)	Model 1^1^	–2.17 (0.49)	<0.001	–1.89 (0.50)	<0.001	–2.03 (0.5)	<0.001	–1.36 (0.50)	0.009
	Model 2^2^	–1.72 (0.50)	0.002	–1.44 (0.52)	0.009	–1.62 (0.51)	0.004	–1.07 (0.51)	0.040
	Model 3^3^	–1.37 (0.51)	0.010	–1.05 (0.53)	0.051	–1.24 (0.52)	0.021	–0.75 (0.52)	0.151
CV (%)	Model 1	–0.30 (0.11)	0.012	–0.34 (0.11)	0.006	–0.31 (0.11)	0.010	–0.34 (0.12)	0.006
	Model 2	–0.23 (0.12)	0.060	–0.32 (0.12)	0.011	–0.28 (0.12)	0.025	–0.33 (0.12)	0.009
	Model 3	–0.27 (0.12)	0.026	–0.37 (0.12)	0.005	–0.33 (0.12)	0.010	–0.37 (0.12)	0.004
J-index	Model 1	–0.85 (0.17)	<0.001	–0.77 (0.17)	<0.001	–0.82 (0.17)	<0.001	–0.51 (0.18)	0.001
	Model 2	–0.68 (0.18)	<0.001	–0.60 (0.18)	0.003	–0.66 (0.18)	0.001	–0.68 (0.18)	0.007
	Model 3	–0.58 (0.18)	0.003	–0.48 (0.18)	0.011	–0.51 (0.18)	0.006	–0.41 (0.18)	0.025
MAGE (mg/dL)	Model 1	–1.25 (0.41)	0.004	–1.3 (0.41)	0.014	–1.3 (0.41)	0.004	–1.22 (0.41)	0.006
	Model 2	–1.00 (0.43)	0.021	–1.11 (0.44)	0.009	–1.06 (0.43)	0.013	–1.10 (0.43)	0.013
	Model 3	–1.10 (0.43)	0.014	–1.22 (0.45)	<0.001	–1.23 (0.44)	0.008	–1.18 (0.43)	0.009
CONGA-1 (mg/dL)	Model 1	–0.55 (0.18)	0.004	–0.57 (0.18)	0.004	–0.61 (0.18)	0.002	–0.50 (0.18)	0.009
	Model 2	–0.50 (0.19)	0.012	–0.56 (0.29)	0.007	–0.57 (0.19)	0.006	–0.50 (0.19)	0.012
	Model 3	–0.56 (0.19)	0.006	–0.64 (0.2)	0.003	–0.69 (0.2)	0.002	–0.57 (0.19)	0.006
MODD (mg/dL)	Model 1	–0.78 (0.15)	<0.001	–0.65 (0.15)	<0.001	–0.76 (0.15)	<0.001	–0.66 (0.15)	<0.001
	Model 2	–0.65 (0.16)	<0.001	–0.51 (0.16)	0.004	–0.64 (0.16)	<0.001	–0.56 (0.16)	0.002
	Model 3	–0.61 (0.16)	<0.001	–0.46 (0.17)	0.009	–0.59 (0.16)	0.001	–0.52 (0.16)	0.004
Log Time >140 mg/dL (%)	Model 1	–0.17 (0.03)	<0.001	–0.14 (0.03)	<0.001	–0.15 (0.03)	<0.001	–0.10 (0.03)	<0.001
	Model 2	–0.15 (0.03)	<0.001	–0.12 (0.04)	<0.001	–0.13 (0.03)	<0.001	–0.09 (0.03)	<0.001
	Model 3	–0.13 (0.03)	<0.001	–0.10 (0.04)	<0.001	–0.11 (0.04)	<0.001	–0.07 (0.03)	<0.001

Abbreviations: AHEI-2010, alternate healthy eating index; aMED, alternate Mediterranean diet; CONGA-1, continuous overall net glycemic action (measured over 1 hour); CV, coefficient of variation; DASH, dietary approaches to stop hypertension; HEI-2020, healthy eating index-2020; MAGE, mean amplitude of glycemic excursion; MODD, mean of daily difference; SD, standard deviation; SE, standard error.

1 Model 1 was adjusted for age, sex, and energy intake.

2 Model 2 was adjusted for model 1 plus smoking status, education level, alcohol intake, hypertension medication, cholesterol-lowering medication, and average steps per day.

3 Model 3 was adjusted for model 2 plus BMI.

4 False discovery rate-adjusted *P* values.

**FIGURE 3:**
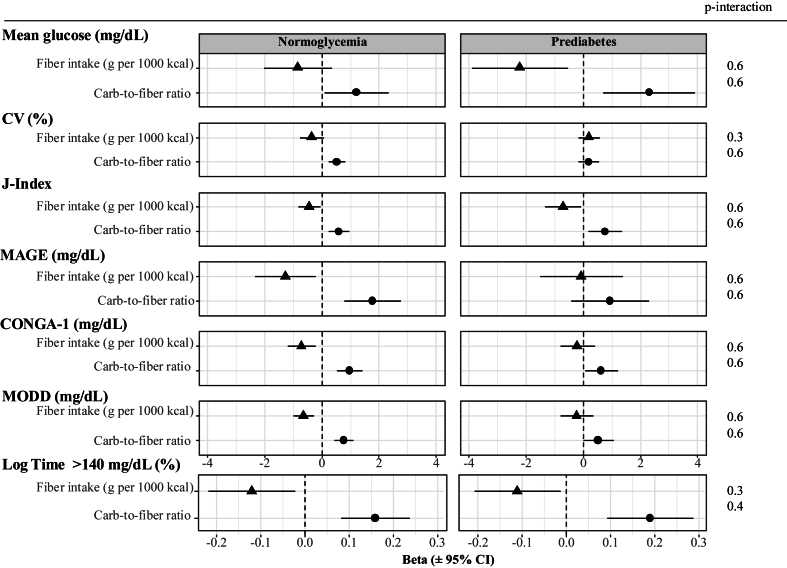
Multivariable linear associations^1^ between carbohydrate quality metrics and CGM-derived measures of glycemic variability stratified by glycemic status (normoglycemia: *n* = 385, prediabetes: *n* = 292). BMI, body mass index; CGM, continuous glucose monitor; CI, confidence interval; CONGA-1, continuous overall net glycemic action (over 1 hour); CV, coefficient of variation; FDR, false discovery rate; MAGE, mean amplitude of glycemic excursion; MODD, mean of daily difference.^1^Model was adjusted for age, sex, energy intake, smoking status, education level, alcohol intake, hypertension medication, cholesterol-lowering medication, average steps per day, BMI. *P*-interactions were false discovery rate-adjusted.

**FIGURE 4:**
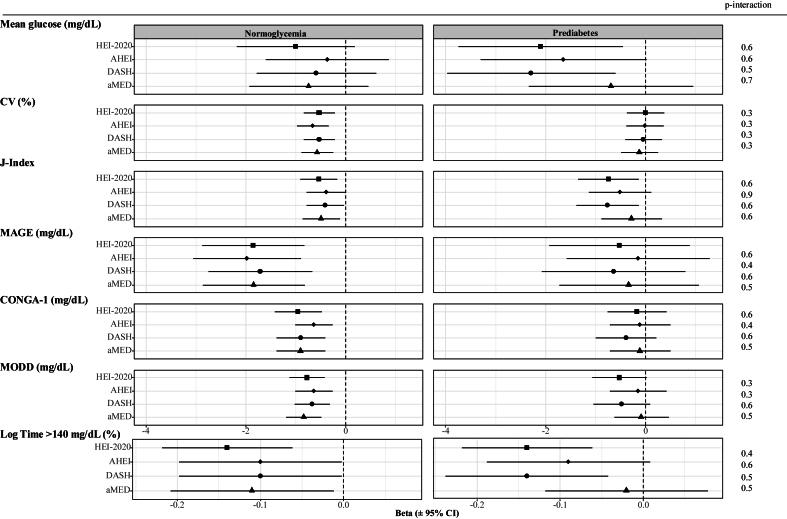
Multivariable linear associations^1^ between diet quality indices and CGM-derived measures of glycemic variability stratified by glycemic status (normoglycemia: *n* = 385, prediabetes: *n* = 292). AHEI, alternate healthy eating index; aMED, alternate Mediterranean diet score; BMI, body mass index; CGM, continuous glucose monitor; CI, confidence interval; CONGA-1, continuous overall net glycemic action (over 1 hour); CV, coefficient of variation; DASH, dietary approaches to stop hypertension; FDR, false discovery rate; HEI-2020, healthy eating index 2020; MAGE, mean amplitude of glycemic excursion; MODD, mean of daily difference. ^1^Model was adjusted for age, sex, energy intake, smoking status, education level, alcohol intake, hypertension medication, cholesterol-lowering medication, average steps per day, BMI. *P*-interactions were false discovery rate-adjusted.

**FIGURE 5:**
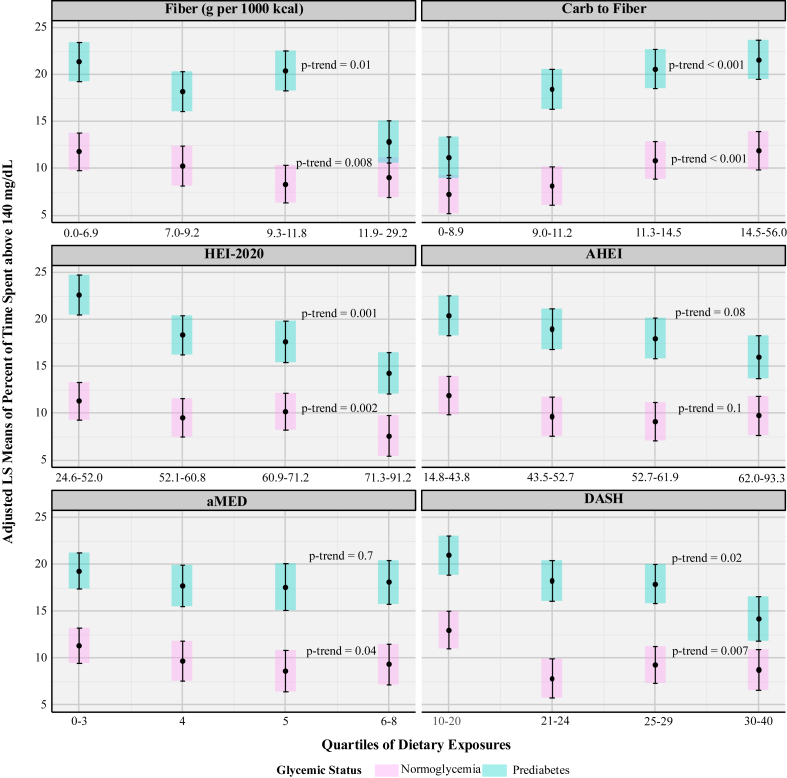
The adjusted least squares means^1^ of percent of time spent >140 mg/dL by quartiles of carbohydrate quality and diet quality metrics by glycemic status (normoglycemia: *n* = 385, prediabetes: *n* = 292). AHEI, alternate healthy eating index; aMED, alternate Mediterranean diet score; DASH, dietary approaches to stop hypertension; HEI-2020, healthy eating index 2020. ^1^The least squares means in this figure were generated from linear regression models with percent of time spent >140 mg/dL not log-transformed. To test the linear trends, regression models with log-transformed percent of time spent >140 mg/dL were employed. Model was adjusted for age, sex, smoking status, education level, alcohol intake, hypertension medication, cholesterol-lowering medication, average steps per day.

We did not observe a statistically significant effect modification by glycemic status in the association between metrics of carbohydrate and diet quality with CGM summary measures (FIGURE 3, FIGURE 4, FIGURE 5). However, when stratified by glycemic status, some associations with CGM metrics of glycemic variability (e.g., MAGE, CONGA, and MODD) became insignificant among those with prediabetes, whereas associations with glycemic burden (mean CGM glucose) were often stronger in those with prediabetes (FIGURE 3, FIGURE 4). Figure 5 shows the adjusted least squares means (±95% confidence interval) of the percent of time spent >140 mg/dL across quartiles of carbohydrate quality and diet quality metrics. We observed a statistically significant trend of decreasing percent of time spent >140 mg/dL across quartiles of measures of carbohydrate quality and most measures of diet quality (except for aMED and AHEI), both among those with normoglycemia and prediabetes. For example, among individuals with prediabetes, consuming a diet with >1 g fiber for every ∼9 g carbohydrates was associated with 7–10% lower time spent >140 mg/dL compared to higher carb-to-fiber ratios (*P*-trend < 0.001). We ran sensitivity analysis excluding individuals below the 1st and above the 99th percentiles of each CGM-derived measure and excluding participants with <3 days of diet records, but the size and significance of associations did not change substantially (data not shown).

## Discussion

In a community-based sample of middle-aged adults without diabetes, we observed that increasing carbohydrate intake at the expense of protein was more consistently associated with higher glycemic variability, suggesting a potentially protective role of protein in glycemic regulation. Associations of higher diet and carbohydrate quality with lower glycemic variability were more pronounced among individuals with normoglycemia. In contrast, associations with lower glycemic burden (mean glucose) were stronger among those with prediabetes.

Currently, no standardized guidelines have been established to determine appropriate thresholds of CGM-derived glycemic traits for T2D prevention among those with normoglycemia or prediabetes. However, one study, involving 499 individuals without diabetes at baseline, demonstrated that a higher baseline time >140 mg/dL was significantly associated with T2D development over 5 y follow-up [38]. CGM sensors have recently become available for purchase over the counter, without a prescription from Abbott and Dexcom. Both new devices use time in a tight range (70–140 mg/dL) as the target goal for individuals without diabetes, so it may be notable that we observed a strong association of the carb-to-fiber ratio with time spent out of this range, especially among individuals with prediabetes.

Thus far, CGMs have been utilized in clinical research to predict glycemic response to food, promote behavioral change, and enhance glycemic management across individuals with normoglycemia to those with T2D [39]. Trials such as the personalized responses to dietary composition (PREDICT) have indicated that meal macronutrient composition and timing contribute to the CGM-derived postprandial glycemic response among healthy adults [40]. However, fewer large-scale observational studies have incorporated CGMs among individuals with normoglycemia or prediabetes to understand the glycemic landscape and investigate the association between diet and CGM-derived measures of glycemic variability. The few studies conducted to date have focused on macronutrient composition, rather than quality, as we have chosen to assess. Higher glycemic variability has been found to be positively correlated with various cardiometabolic risk factors; as such, identifying unique aspects of diet is important in understanding the determinants of dynamic fluctuations in glucose [16]. Keshet et al. [16] found that a moderate to high carbohydrate intake (median %EI = 42.2%) was positively correlated with CGM-derived measures, including CV, J-index, MAGE, and MODD, after adjusting for age and sex, among 7000 adults without diabetes. However, the authors did not consider if the underlying glycemic status modified the observed associations, nor did they assess carbohydrate quality, or overall diet quality and other important covariates. We observed that in individuals with normoglycemia, fiber intake, carb-to-fiber ratio, and diet quality indices (HEI-2020, AHEI, DASH, and aMED) were more strongly associated with CGM metrics, CV, J-index, MAGE, CONGA1, and MODD, compared to those with prediabetes, in whom these associations were weaker and mostly lost their significance. We also reported significant associations between measures of carbohydrate quality and diet quality with the log-transformed percent of time s >140 mg/dL.

Carbohydrate quality and diet quality are important dietary attributes to consider in those with prediabetes. Our findings suggest that these dietary exposures were more strongly associated with CGM mean glucose in prediabetes, compared to normoglycemia, even after accounting for other lifestyle factors and overall adiposity. Our findings are consistent with those of a smaller cross-sectional study including 53 individuals with impaired glucose tolerance (based on oral glucose tolerance test) which observed that CGM mean glucose, CONGA-1 and J-index was lower in those with higher carbohydrate quality (consuming more whole grains and less refined grains); however, these observations were not present in their sample of participants with normoglycemia [17]. In our large study and in another very small study [41], similar associations were only observed among normoglycemic participants.

Comparison across studies is challenging for many reasons, including the different CGM sensors, population characteristics, and variation in food consumption patterns from feeding in intervention studies to free-living observational data. In Keshet et al. [16], using Abbott FreeStyle Libre sensor, the reference ranges of CGM measures [Mean (SD) of J-index: 12.03 (3.0), MAGE: 24.02 (6.45), MODD: 12.7 (3.4), CV: 15.65 (3.63)] were lower than our study, even among our individuals with normoglycemia, which may be partially due to older age, higher BMI, differences in lifestyle and dietary habits in our population, or use of different CGM sensors. Another study by Freckmann et al. [42], also using the FreeStyle Libre, investigated the pre and postprandial glucose profiles of 36 young adults under fixed meal timing and everyday life conditions, reporting lower mean glucose values compared to our study which may be attributable to difference in age distribution and adjustment of CGM values to capillary plasma-equivalent glucose values.

As expected, in FHS, we observed higher values for mean glucose and metrics of glycemic variability among individuals with prediabetes than those with normoglycemia, which is in line with prior studies [[43], [44], [45]]. We also recently reported the physiological ranges of time spent in various CGM glucose concentrations in >1000 FHS participants without diabetes, showing that individuals with prediabetes spent substantially more time each day with CGM glucose >140 mg/dL (>5 hours/day), but those with normoglycemia still spend ∼3 hours/day >140 mg/dL, on average [46]. Earlier investigations of younger, healthier adults (using Dexcom G6 pro, but with daily manual calibration) had suggested that individuals without diabetes spend the majority of time within a tight range of 70–140 mg/dL and therefore may exhibit limited variation in CGM metrics [47]. It is possible that the study by Dimova et al. [17] (using the FreeStyle Libre) did not observe associations of diet with CGM metrics in individuals with normoglycemia due to their lower CGM metric values, limiting the ability to capture how diet is affecting glucose fluctuations (e.g., mean CGM glucose was <100 mg/dL compared with our normoglycemic participants with mean CGM glucose =113.7 mg/dL) [17]. Despite the reported associations in our study, whether glycemic variability mediates the longitudinal association between carbohydrate quality and incidence of T2D or T2D-related health outcomes has yet to be determined.

Poor carbohydrate quality and diet quality are well-established risk factors for T2D development and progression. In this study, we showed that fiber intake and the ratio of carbohydrate to total fiber intake were associated with worsened glycemic variability. A prior investigation in the Framingham offspring cohort has indicated that replacing refined grains with whole grains was associated with lower cardiometabolic risk over time [48]. A previous cohort study of United States females demonstrated that a higher total fiber intake (grams/day) was associated with a 20% reduction in risk of T2D and a higher carb-to-fiber ratio was associated with a 9% increase in risk of T2D [49]. Other observational studies have reported similar results with regard to protective associations of higher fiber intake and lower carb-to-fiber ratio on longitudinal changes in abdominal obesity [21], prevalent metabolic syndrome [50], and incident coronary artery disease [51]. Lastly, a recent meta-analysis of 10 prospective cohort studies indicated that high glycemic index and glycemic load, 2 markers of carbohydrate quality, were associated with a 15% increased T2D and CVD incidence [9]. Nutrient substitution models (i.e., replacing 5% energy from carbohydrates with proteins or fats) provide a statistical framework to investigate the health effects of macronutrient composition while holding total EI constant [34]. Given that for an individual, the total caloric intake is relatively constrained [34], substitution analysis has been commonly used in nutritional epidemiology to mimic feeding trials [52]. In the context of our study, a 5% energy substitution corresponds to replacing ∼25 g of carbohydrate (4 kcal/g) with either 25 g of protein (4 kcal/g) or ∼11 g of fat (9 kcal/g) in a 2000 kcal diet.

In the present study, we found that higher adherence to diet quality indices was associated with lower mean glucose concentrations and lower glycemic variability. The association between diet quality, T2D, and related cardiometabolic outcomes has been extensively studied [6,53,54]. A systematic review and meta-analysis of 113 cohort studies suggested that greater adherence to diet quality indices, HEI, AHEI-2010, and DASH, is associated with a 12%, 20%, and 22% reduction in incident T2D, respectively[6]. Similarly, a higher Mediterranean diet score was associated with a 17% reduction in the risk of T2D [55]. In our stratification analysis, we observed that higher diet quality was associated with lower glycemic variability, mainly among those with normoglycemia. Despite higher EI and higher BMI, individuals with prediabetes had similar diet quality than those with normoglycemia, possibly due to a lack of lifestyle modifications to address the underlying pathology. Losing 5%–10% of body weight and increased physical activity are pillars of lifestyle modifications to reverse prediabetes and prevent T2D [56].

Our study has several limitations. ASA24 is a valid and reliable tool for detailed dietary assessment [57], yet the collected data may not reflect dietary intake during all CGM days. It is possible that during the CGM wear time, participants changed their behavior, modified their diet and physical activity levels, which could alter their glycemic profile. Although we accounted for average daily steps counts, which was objectively measured from Fitbit devices, we must acknowledge that step count provides a general measure of activity volume [58], but may not fully capture the complexity, such as intensity, duration, timing relative to meals, or resistance training, all of which may differentially influence glycemic variability. Although we have taken various potential confounders into account, we cannot rule out the possibility of residual confounding. Further, our study population is predominantly comprised of White individuals of European descent, residing mainly in one geographic location, which may reduce the generalizability of the findings. In this study, participants who declined to wear CGM may have different demographic characteristics from those who did not, which may have led to selection bias. Lastly, we do not have follow-up data for our participants yet as our data collection is ongoing; therefore, we cannot evaluate how habitual dietary intake affects glycemic variability and long-term health outcomes, including but not limited to T2D. The strengths of this study are the large sample size and the inclusion of both individuals with normoglycemia and those with prediabetes. Through providing a comprehensive analysis of various carbohydrate and diet quality metrics, this study contributes to the ongoing endeavors characterizing variations in CGM glucose. Although CGM can be used as an adjuvant to behavioral lifestyle interventions that aim to prevent T2D [59], our results are consistent with the notion that increasing fiber intake to the recommended levels, and adhering to dietary guidelines, is essential to reduce glycemic variability among individuals with prediabetes.

In conclusion, high carbohydrate intake and low carbohydrate quality, in addition to poor diet quality, are associated with unfavorable CGM metrics including mean glucose and measures of glucose variability. Further large-scale epidemiologic studies that consider other lifestyle factors, such as phsyical activity and sleep, and randomized controlled trials are needed to confirm these findings.

## Author contributions

The authors’ responsibilities were as follows – BB, HL, VV, AG, DWS, JMM, NMM, MEW, NLS: designed research; BB, NS, DF: conducted research; BB: analyzed data and wrote the paper; NLS: had primary responsibility for all content; and all authors: read and approved the final manuscript.

## Data availability

Data described in the manuscript, code book, and analytic code will be made available upon request pending.

## Funding

This investigation was supported by the Framingham Heart Study’s National Heart, Lung, and Blood Institute contracts (N01-HC25195, HHSN268201500001I, and 75N92019D00031) with additional support from the National Institute of Diabetes and Digestive and Kidney Diseases R01DK129305. Dexcom also provided continuous glucose monitors at a discounted rate.

## Conflict of interest

NLS reports financial support was provided by National Institute of Diabetes and Digestive and Kidney Diseases. JMM reports financial support was provided by National Heart Lung and Blood Institute. NLS and DWS received funding for an investigator-initiated research grant from Novo Nordisk, unrelated to the current project. All other authors report no conflicts of interest.
