# Supplementary material for: Associations of diet composition and quality with continuous glucose monitor-derived glycemic metrics in a community-based cohort
Source: Am J Clin Nutr. 2025 Jul 31;122(4):942–53. doi: 10.1016/j.ajcnut.2025.07.026 (PMC12495531; doi:10.1016/j.ajcnut.2025.07.026)
Supplement: Multimedia component 1 [file mmc1.docx]

**Supplementary Figure 1.** Study flow chart.

Framingham Heart Study Generation 3, Omni 2, New Offspring Spouse Cohorts

(n=1291)

Participants with ≥3 days of CGM data

(n=1037)

Participants with valid CGM and Fitbit data who filled out ASA24

(n=851)

Participants with valid CGM, Fitbit, and diet data ASA24

(n=782)

Participants with without diabetes

(n=701)

Exclude those:

–with less than two days of dietary records

(n= 60)

–with implausible energy intake (females: <600, >4400 kcals/day; males: <650, >5700 kcals/day) (n= 8)

– with extreme portions of a food consumed at one eating occasion (n=1)

Excluding those who did not have any diet data (n=186)

Exclude those with T2D or taking glucose- lowering medications (n=81)

Eligible study sample

(n=677)

Exclude those with missing information on important covariates (average steps, n=23 and education n=1)

Exclude those:

–who refused to wear the device (n=108)
–who did not return the CGN device (n=76)
–with the CGM devices that had no data (n=29)
­­–those with CGM devices that had data,

but no complete days (n=20)
–those with CGM devices that had 1-2 days (n=27)

Of those who did not have any dietary data (n=186), 38 individuals had diabetes and 8 individuals had missing information on important covariates (n=6).

**Supplementary Table 1.** Participant characteristics of those who did not complete any diet data and with no missing values on important covariates (n=138)

|  | Total population | Individuals  with normoglycemia |  | Individuals  with prediabetes |
| --- | --- | --- | --- | --- |
| No. of participants | 138 | 68 |  | 70 |
| Characteristics^1^ |  |  |  |  |
| Sex, female | 75 (54.3) | 44 (64.7) |  | 31 (44.3) |
| Age, years | 60.3 (9.2) | 59.2 (9.9) |  | 61.4 (8.4) |
| Race, White or Caucasian | 118 (85.5) | 60 (88.2) |  | 58 (82.8) |
| Current smoker | 15 (10.8) | 9 (13.2) |  | 6 (8.5) |
| HbA1c | 5.3 (0.4) | 5.2 (0.3) |  | 5.5 (0.4) |
| Hypertension medication | 44 (31.9) | 16 (23.5) |  | 28 (40) |
| lipid-lowering medication | 52 (37.7) | 19 (27.9) |  | 33 (47.1) |
| BMI, kg/m2 | 27.9 (5.3) | 26.1 (5.06) |  | 29.6 (4.9) |
| Steps, average/d | 9310 (4595) | 9601 (4697) |  | 9030 (4514) |
| Glycemic traits^1^ |  |  |  |  |
| Mean (mg/dl) | 120.4 (15.1) | 115.9 (13.1) |  | 124.7 (15.7) |
| CV (%) | 15.3 (3.2) | 15.1 (3.2) |  | 15.5 (3.2) |
| MAGE (mg/dl) | 41.4 (11.7) | 39.9 (10.1) |  | 42.7 (13.0) |
| CONGA-1 (mg/dl) | 22.04 (5.6) | 21.5 (5.3) |  | 22.6 (5.9) |
| J-index | 20.1 (5.5) | 18.4 (4.1) |  | 21.7 (6.1) |
| MODD (mg/dL) | 18.7 (4.8) | 17.4 (3.9) |  | 19.9 (5.4) |
| Time above 140 mg/dL (%) | 17.1 (15.2) | 12.2 (10.1) |  | 23.2 (19.2) |

^1^Mean (SD) or n (%)

HbA1c = hemoglobin A1c, BMI = Body mass index CGM = continuous glucose monitor, CV = coefficient of variation, MAGE = mean amplitude of glycemic excursions, CONGA-1 = continuous overall net glycemic action (measured over 1 hour), MODD = mean of daily differences.

The healthy eating index (HEI-2020) assesses adherence to 13 components corresponding to the 2020-2025 dietary guidelines for Americans (DGA), including 9 adequacy components, total fruits, whole fruits, total vegetables, greens and beans, whole grains, dairy, total protein foods, seafood and plant proteins, and fatty acids, with increased intakes resulting in a greater diet quality score, and 4 moderation components, refined grains, sodium, added sugars, and saturated fats, with increased intakes reducing the diet quality score (1).

**Supplementary Table 2.** Median scores [population min, population max] of the healthy eating index (HEI-2020) components according to glycemic status.

| Diet quality | Total population | Normoglycemia | Prediabetes |
| --- | --- | --- | --- |
| HEI-2020 |  |  |  |
| Total Fruits | 2.6 [0, 5] | 2.7 [0, 5] | 2.6 [0, 5] |
| Whole Fruits | 4.1 [0, 5] | 4.6 [0, 5] | 4.1 [0, 5] |
| Total Vegetables | 4.2 [0, 5] | 4.3 [0, 5] | 4.2 [0, 5] |
| Greens and Beans | 4 [0, 5] | 4.3 [0, 5] | 4.0 [0, 5] |
| Whole Grains | 2.9 [0, 10] | 2.9 [0, 10] | 2.8 [0, 10] |
| Dairy | 5.4 [0, 10] | 5.4 [0, 10] | 5.6 [0, 10] |
| Total Protein Foods | 5 [0, 5] | 5 [0.0, 5] | 5 [1.3, 5] |
| Seafood and Plant Proteins | 5 [0, 5] | 5 [0, 5] | 5 [0, 5] |
| Fatty Acids | 4.9 [0, 10] | 5.1 [0, 10] | 4.5 [0, 10] |
| Refined Grains | 8.5 [0, 10] | 8.3 [0, 10] | 8.7 [0, 10] |
| Sodium | 4.6 [0, 10] | 4.7 [0, 10] | 4.6 [0, 10] |
| Added Sugar | 9.3 [0, 10] | 9.4 [0, 10] | 9.4 [0, 10] |
| Saturated Fat | 4.8 [0, 10] | 5.3 [0, 10] | 4.5 [0, 10] |

HEI-2020= healthy eating index.

Of the 11 alternate healthy eating index (AHEI) components, 6 components were positively scored, including vegetables (excluding potatoes), fruit, whole grains, nuts, legumes, and vegetable protein, long-chain (omega-3) fats, and polyunsaturated fatty acids and 5 components are negatively scored, including red and processed meat, sugar-sweetened beverages (SSBs) and fruit juice, *trans* fat, sodium, and alcohol (2). Since ASA24 output does not include information on *trans* fat intake, in addition to the SSBs and fruit juice component, we removed the *trans* fat category from the scoring, and obtained the SSBs and fruit juice component through adding relevant USDA food and nutrient database for dietary studies (FNDDS) food codes.

**Supplementary Table 3.** Median scores [population min, population max] of the alternate healthy eating index (AHEI) components according to glycemic status.

| Diet quality | Total population | Normoglycemia | Prediabetes |
| --- | --- | --- | --- |
| AHEI-2010 |  |  |  |
| Vegetables | 4.8 [0, 10] | 4.8 [0, 10] | 4.8 [0, 10] |
| Fruit | 1.6 [0, 10] | 1.6 [0, 10] | 1.6 [0, 10] |
| Whole Grains | 2.8 [0, 10] | 2.9 [0, 10] | 2.6 [0, 10] |
| Nuts and Legumes | 10 [0, 10] | 10 [0, 10] | 10 [0, 10] |
| Long-Chain Omega-3 Fats | 1.8 [0, 10] | 1.8 [0, 10] | 1.9 [0, 10] |
| PUFA | 8.4 [0.4, 10] | 8.5 [1.4, 10] | 8.2 [0.4, 10] |
| SSBs and Fruit Juice | 10 [0, 10] | 10 [0, 10] | 10 [0, 10] |
| Red and Processed Meat | 6.3 [0, 10] | 7.1 [0, 10] | 5.3 [0, 10] |
| Sodium | 5 [0, 10] | 5 [0, 10] | 5 [0, 10] |
| Alcohol | 2.5 [0, 10] | 2.5 [0, 10] | 2.5 [0, 10] |

AHEI-2010= alternate healthy eating index , SSBs=sugar-sweetened beverages, PUFA= polyunsaturated fatty acids.

The dietary approaches to stop hypertension (DASH) components include vegetables (excluding potatoes), nuts, seeds, legumes, whole grains, fruits, and low-fat dairy. For these components, the highest quintile received 5 points, and the lowest quintile received 1 point. Conversely, for red and processed meat, SSBs, and sodium, the scoring was reversed. We calculated the SSBs and low-fat dairy components through summing the relevant USDA FNDDS food codes (3). In our population, the consumption of SSBs and low-fat dairy were particularly low, therefore, for low-fat dairy, the first 3 quintiles were scored as 1, and for SSBs, the first 3 quintiles were scored as 5.

**Supplementary Table 4.** Median scores [population min, population max] of the dietary approaches to stop hypertension (DASH) components according to glycemic status.

| Diet quality | Total population | Normoglycemia | Prediabetes |
| --- | --- | --- | --- |
| DASH |  |  |  |
| Whole Fruit and Fruit Juice | 3 [1, 5] | 3 [1, 5] | 3 [1, 5] |
| Vegetable | 3 [1, 5] | 3 [1, 5] | 3 [1, 5] |
| Nuts and Legumes | 3 [1, 5] | 3 [1, 5] | 3 [1, 5] |
| Whole Grains | 3 [1, 5] | 3 [1, 5] | 3 [1, 5] |
| Low fat dairy | 1 [1, 5] | 1 [1, 5] | 1 [1, 5] |
| Sodium | 3 [1, 5] | 3 [1, 5] | 3 [1, 5] |
| Red and Processed Meat | 3 [1, 5] | 3 [1, 5] | 3 [1, 5] |
| SSBs and Fruit Juice | 5 [1, 5] | 5 [1, 5] | 5 [1, 5] |

DASH= dietary approaches to stop hypertension, SSBs=sugar-sweetened beverages.

The alternate Mediterranean diet (aMED) includes 9 components, vegetables (excluding potatoes), fruits, nuts, whole grains, legumes, fish, monounsaturated-to-saturated fat ratio, consumption above the median received 1 point. For red and processed meat intake below the median received 1 point. Additionally, alcohol consumption between the ranges of 5-15 g/day for women and 15-25 g/day for men received 1 point (4). The aMED components are scored binary, and therefore, the supplementary table cannot be provided.

Reference:

1. Shams-White MM, Pannucci TE, Lerman JL, Herrick KA, Zimmer M, Mathieu KM, et al. Healthy eating index-2020: review and update process to reflect the dietary guidelines for Americans, 2020-2025. Journal of the Academy of Nutrition and Dietetics. 2023;123(9):1280-8.

2. Chiuve SE, Fung TT, Rimm EB, Hu FB, McCullough ML, Wang M, et al. Alternative dietary indices both strongly predict risk of chronic disease. J Nutr. 2012;142(6):1009-18.

3. Rhodes DG, Morton S, Myrowitz R, Moshfegh AJ. Food and Nutrient Database for Dietary Studies 2019–2020: An application database for national dietary surveillance. Journal of Food Composition and Analysis. 2023;123:105547.

4. Fung TT, Hu FB, McCullough ML, Newby PK, Willett WC, Holmes MD. Diet Quality Is Associated with the Risk of Estrogen Receptor–Negative Breast Cancer in Postmenopausal Women1. The Journal of Nutrition. 2006;136(2):466-72.
